# Chondromodulin-1 directly suppresses growth of human cancer cells

**DOI:** 10.1186/1471-2407-9-166

**Published:** 2009-05-31

**Authors:** Hisashi Mera, Hiroyuki Kawashima, Tatsuya Yoshizawa, Osamu Ishibashi, Md Moksed Ali, Tadashi Hayami, Hiroshi Kitahara, Hiroshi Yamagiwa, Naoki Kondo, Akira Ogose, Naoto Endo, Hiroyuki Kawashima

**Affiliations:** 1 Division of Orthopedic Surgery, Niigata University Graduate School of Medical and Dental Sciences, Niigata, Japan; 2 Division of Cell Biology and Molecular Pharmacology, Niigata University Graduate School of Medical and Dental Sciences, Niigata, Japan

## Abstract

**Background:**

Chondromodulin-1 (ChM1), an endogenous anti-angiogenic factor expressed in cartilage, has been suggested to inhibit invasion of endothelial cells into cartilage. In addition, the ectopic administration of ChM1 has been reported to suppress tumorigenesis *in vivo*. However, it is unclear whether the anti-tumor effect is due to not only the anti-vascularization effect of ChM1, but also its direct action against oncocytes. In the present study, we sought to determine whether ChM1 has a direct action on tumor cells.

**Methods:**

BrdU incorporation assay was performed on human umbilical vein endothelial cells (HUVECs), normal human dermal fibroblasts (NHDFs), HepG2 cells and HeLa cells in the presence or absence of recombinant human ChM1 (rhChM1). An adenovirus that expresses ChM1, Ad-ChM1, was established and applied to the tumor xenografted *in vivo*, and to *in vitro *tumor cells cultured on plates or in soft agar. Cell cycle-related proteins and the phosphorylation of Erk, Akt, and GSK3β, the downstream molecules of the extracellular matrix-integrin signaling pathways, in HepG2 cells treated with or without Ad-ChM1 were detected by western blot analysis. Luciferase reporter assays of STAT, GAS, and ISRE, which participate in another cytokine signaling pathway, ware performed in HepG2, HeLa, and HUVEC cells.

**Results:**

ChM1 suppressed BrdU incorporation in HUVECs and in HepG2 cells dose-dependently, but did not suppress BrdU incorporation in NHDFs and HeLa cells cultured on plates. In soft agar, however, ChM1 suppressed the growth of HeLa cells, as well as HepG2 cells. Western blot analyses demonstrated that ChM1 decreased the levels of cyclin D1, cyclin D3, and cdk6 and increased those of p21^cip1 ^without affecting the phosphorylation levels of Erk, Akt, and GSK3β in HepG2 cells. The luciferase reporter assay demonstrated that ChM1 suppressed the transcriptional activities of STAT and GAS but not of ISRE.

**Conclusion:**

ChM1 directly suppressed the proliferation of tumor cells in an anchorage-independent manner. However, ChM1 did not alter the phosphorylation of downstream molecules, at which the signaling pathways through growth factor and cytokine receptors converge with the anchorage-dependent pathway. Our results show that ChM1 has a direct anti-tumor effect; moreover, this effect occurs by inhibiting the STAT signaling pathway.

## Background

Malignant tumor cells produce various growth factors that induce angiogenesis to supply nutrition for their own growth. Thus molecules that inhibit angiogenesis are good candidates for anti-tumor agents [1]. Indeed, some studies in which angiogenesis was targeted have provided encouraging results. Recently, however, it was reported that monotherapy with the monoclonal antibody bevacizmab, which targets vascular endothelial growth factor (VEGF), or an endogenous anti-angiogenic agent such as endostatin produced only moderate suppression of tumor growth compared to a combined therapy that included a cytotoxic agent [2,3]. These observations suggest that a molecule with both cytotoxic and anti-angiogenic activities may have a stronger anti-cancer effect. However, such a molecule has not been identified.

Chondromodulin-1 (ChM1) is a 25 kDa glycoprotein that is expressed mainly in cartilage. ChM1 shows anti-angiogenic activity and has been suggested to inhibit endothelial cells from invading cartilage [4-6]. Recently, we reported that the ectopic administration of ChM1 dramatically suppresses tumorigenesis *in vivo *[7], which suggests that ChM1 acts directly against tumor cells. ChM1 can have either a positive or negative effect on cell proliferation: It promotes the proliferation of chondrocytes and osteoblasts [4,6,8], but suppresses growth of endothelial cells and T-cells [5,9].

ChM1 also promotes anchorage-independent growth of chondrocytes [6]. Anchorage-independent growth is a characteristic of non-adherent cells, including oncocytes [10], chondrocytes [11-14], and hemocytes [15,16]. On the other hand, transforming growth factor β (TGFβ) also modulates cell growth both positively and negatively. TGFβ promotes anchorage-independent growth of chondrocytes [6,11,12], but suppresses or promotes anchorage-independent growth of tumor cells depending on the type and state of the cells [14,17-19]. Thus, ChM1 may also suppress tumor cell growth.

Anchorage-dependent signaling involves extracellular matrix-integrin complexes and their downstream molecules such as Erk, Akt, and GSK3β, which are shared with the signaling pathway activated by cytokine receptor stimulation [10,20-22]. Abnormality in this signaling pathway, of tumor suppressor proteins, or a combination of both, constitutively activates oncocytes, thereby inducing anchorage-independent tumor growth. The cytokine signaling pathway involving the Signal Transducers and Activators of Transcription (STAT) protein [3,23-27], a latent transcriptional factor activated by the Janus Kinase (JAK) family of tyrosine kinase, is also modified in various types of tumor cell. Therefore, the STAT signaling pathway may also be involved in the putative action of ChM1.

The aim of the present study was to determine whether ChM1 has a direct action on tumor cells. Here, we report that ChM1 directly suppresses tumor cell anchorage-independent growth by inhibiting the anchorage-independent STAT signaling pathway.

## Methods

### Reagents and antibodies

Anti-ChM1 polyclonal antibody, kindly provided by Dr. Hiraki (Department of Molecular Interaction and Tissue Engineering, Institute for Frontier Sciences, Kyoto University, Kyoto, Japan), was used for western blot analysis [28]. Other primary antibodies were purchased from Cell Signaling Technology Inc (Boston, Massachusetts,) to detect cell-cycle related proteins, Erk (p44/p42), Akt, GSK3β, and their phosphorylated forms. The constructs of pSTAT RE-TK hRluc (STAT-luc), pISRE RE-TK hRluc (ISRE-luc), and pGAS RE-TK hRluc (GAS-luc), provided by Dr. Yokoyama K., and obtained from RIKEN BioResource Center, Tsukuba, Japan, were used for the luciferase reporter assays.

### Cell culture

HEK 293, HepG2 (human hepatocellular carcinoma), HeLa (human uterine cervical adenocarcinoma), and PC-3 (human prostate adenocarcinoma) cells were obtained from the American Type Culture Collection (Rockville, MD). The human osteosarcoma cell line, NOS-1, which is osteoid inducible in xenografted tumors in nude mice, was established previously from a 16 year-old male Japanese patient [29,30]. HepG2 and HeLa cells were cultured in DMEM (Gibco/Invitrogen, Grand Island, NY), PC-3 cells in Ham's F12K (Sigma-Aldrich, Inc., St. Louis, MO), and NOS-1 cells in RPMI (Gibco) supplemented with 10% fetal bovine serum (FBS) at 37°C under 5% CO_2 _in air. Human umbilical vein endothelial cells (HUVECs) and normal human dermal fibroblasts (NHDFs) were obtained commercially (Cambrex, Walkersville, MD). HUVECs were grown in EGM2 medium (EBM complete medium with supplements; Sanko-Junyaku, Tokyo, Japan), and NHDFs in FGM2 medium (FBM medium with supplements; Sanko-Junyaku) at 37°C under 5% CO_2 _in air. Cells were used at passages 2 through 4 after acquisition.

### DNA synthesis assay (BrdU incorporation)

HUVECs and NHDFs were harvested with trypsin/EDTA and suspended in EGM2 and FGM2 as appropriate. The cells were seeded at 3 × 10^4 ^cells/ml into a 96-well multi titer plate (100 μl/well) and cultured for 24 hours. The cells were then starved in 0.5% FBS containing Opti-MEM for 12 hours and stimulated with 10 ng/ml FGF-2 (PeproTech EC, London, UK) in either the presence or absence of 25 μg/ml rhChM1 for another 24 hours. Cells were labeled with BrdU during the last 3 hours of this incubation. HepG2 cells were harvested with trypsin/EDTA and suspended at a density of 5 × 10^3 ^cells/ml in 10% FBS containing DMEM. HeLa cells were harvested similarly and suspended at a density of 6 × 10^4 ^cells/ml. Cells were then seeded into a 96-well multi titer plate (100 μl per well), and cultured for an additional 36 hours. The medium was replaced with one containing either 10 μg/ml or 25 μg/ml rhChM1, BrdU was added, and the cells were cultured for 6, 12 or 24 hours. BrdU incorporation by the cells was measured at least in triplicate at each time point using a cell proliferation ELISA BrdU colorimetric kit according to the manufacturer's instructions (Roche, Basel, Switzerland). Absorbances at 450 nm, referenced at 655 nm, were measured using a Model 680 Microplate Reader (Bio-Rad, Hercules, CA)

### Adenovirus preparation

The human ChM1 cDNA expression vector (pcDNA3-ChM1) was provided by Dr. Hiraki (Department of Molecular Interaction and Tissue Engineering, Institute for Frontier Sciences, Kyoto University, Kyoto, Japan). This cDNA was inserted into a cassette cosmid carrying an adenovirus type-5 genome lacking the E1A, E1B and E3 regions, and in which the Swa I cloning site is flanked by the CAG promoter at the 5' end and by a rabbit globin poly (A) sequence at the 3' end (Takara Bio Inc., Shiga, Japan). In 293 cells, recombination between the homologous regions of the linearized transfer cosmid vector and the adenovirus genome resulted in formation of the complete adenoviral recombinant that contains the ChM1 cDNA. Before use in experiments, the adenovirus was purified by sequential centrifugation in double CsCl step gradients as previously described [31]. Titers of viral stocks were determined by a plaque assay of 293 cells. Viral suspensions were stored at -80°C. The virus was thawed on ice prior to use.

### Adenovirus treatment in vivo

Six- to eight-week old BALB/c athymic nude mice (Clea Japan Inc., Tokyo, Japan) were used. Animal experiments were performed in accordance with the institutional guidelines of the university committee on the use and care of animals. Mice were inoculated with 5 × 10^6 ^HepG2 cells in the flank and tumors were allowed to grow to a volume of 150 mm^3^. Animals were divided into three treatment groups: Ad-ChM1 injection (n = 6); Ad-LacZ injection (n = 6); and injection of control vehicle (Phosphate-Buffered Saline, PBS) (n = 6). Adenovirus vectors (1 × 10^8 ^plaque forming units/100 μl) were injected directly into the foci center on days 0, 2 and 4 of treatment. Tumor length and width were measured with calipers over a period of five weeks. Tumor volume was calculated as (length × width^2^)/2.

### Counting the number of total cells and viable cells in vitro

Approximately 0.5~2.5 × 10^4 ^cells were plated onto 35 mm culture plates and cultured for 24 hrs. Cells were then infected with Ad-ChM1 or Ad-LacZ as a control, at an appropriate multiplicity of infection (MOI) and were further cultured. The MOI for each cell line was selected to produce the optimum effect of ChM1 without cytotoxicity by Ad-LacZ. The total number of cells was counted using a hemocytometer at 24 hrs, 36 hrs, 48 hrs, and 72 hrs after infection with adenoviruses. Viable cells were identified using the trypan blue exclusion method and were counted at each sampling interval. These experiments were carried out at least in triplicate.

### Anchorage-independent growth assay

HepG2 and HeLa cells were cultured on 35 mm culture plates and infected with adenoviruses as described above. Six hours (6 hrs) after adenovirus infection, colony formation assays were performed. The cells were detached and suspended in a culture medium containing 0.68% malting agar (Sigma-Aldrich). The cell suspension was then plated on culture medium containing 0.4% agarose that had been allowed to harden beforehand. The cells were cultured in a volume of 300 μl for 21 days with changes to fresh medium every 3 to 4 days. The numbers and sizes of the colonies were measured under a phase contrast microscope on days 7, 14, and 21 of culture. The experiment was carried out in triplicate.

### Western blotting

To detect ChM1 and cell-cycle related proteins, the culture medium was collected and subjected to trichloroacetic acid (TCA) precipitation. The pellet produced by TCA precipitation was resolved in RIPA buffer containing a protease inhibitor cocktail (Sigma-Aldrich) and PMSF (500:1:1). For whole cell extracts, cells were scraped, lysed with RIPA buffer, and the lysate diluted with an equal volume of buffer containing 2-mercaptoethanol. Xenografted tumor specimens were harvested 48 hrs after adenoviral infection, followed by homogenization in lysis buffer (8 M Urea, 0.2% sodium dodecyl sulfate, 0.8% Triton X-100, 3% 2-mercaptoethanol). Insoluble materials were removed by centrifugation. The supernatants were boiled for 3 min and stored at -20°C. For SDS-PAGE, proteins (10~80 μg/lane) were run on 8~15% polyacrylamide slab gels and transferred to polyvinylidene difluoride membranes. The membranes were blocked with 5% skimmed milk in TBS with 0.1% Tween 20 (TBS-T) for 1 hr at room temperature, and incubated overnight at 4°C with primary antibodies diluted at 1:1000 to 1:4000. After washing at least twice in TBS-T, the membranes were incubated with a horseradish peroxidase-conjugated secondary antibody (diluted 1:25000 with TBS-T) at room temperature for 1 hour. The membrane was washed twice with TBS-T and immunoreactivity was visualized using the Immobilone western blotting detection system (Millipore, Billerica, MA).

Films of cell-cycle related proteins were developed and scanned, and bands were analyzed as a ratio of target protein/α-tubulin control using the Scion Image for Windows program, version 4.02.

### Luciferase reporter assay

Three reporter constructs were obtained from RIKEN BRC (Tsukuba, Japan). The nucleotide sequences of the response elements were as follows: 5'-gatccagttcccgtcaatcg-3' for STAT, 5'-gatccagaaacaaaaacaag-3' for ISRE, and 5'-gatccttccgggaattctgggaag-3' for GAS. These constructs express Renilla luciferase. We prepared a reference construct by digesting the HSV-TK promoter, between the BamH1 site and Hind III sites, from the pRL-TK vector (Promega Corporation, Madison, WI, USA) that expresses Renilla luciferase, and cloning this fragment into the pGL4.18 [*luc2p/Neo*] vector (Promega) that expresses Firefly luciferase.

Cells were infected with virus and cultured for 12 hours. They were then washed twice with culture medium and then transfected with various luciferase expression vectors by the lipofection method using Fugene-6 (Roche Diagnostics). Twenty-four hours after transfection, the cells were harvested and a Dual-Luciferase TM reporter assay system (Promega) was used for sequential measurement of Firefly and Renilla luciferase activities using the specific substrates beetle luciferin and coelenterazine, respectively. Quantification of luciferase activities and calculation of relative ratios were carried out using a luminometer (TD-20/20, Turner Designs, Sunnyvale, CA). In these experiments, at least three independent transfections were performed.

### Statistical analysis

Student's *t*-test was performed for group comparisons of BrdU incorporation on HUVECs and NHDFs. Fisher's protected least significant difference procedure was performed after repeated-measures analysis of variance (ANOVA) for group comparisons of BrdU incorporation on HepG2 and HeLa cells, and for the comparison of luciferase reporter assays on HepG2, HeLa, and HUVECs.

## Results

### Recombinant human ChM1 suppresses DNA synthesis in some tumor cells

**Figure 1:**
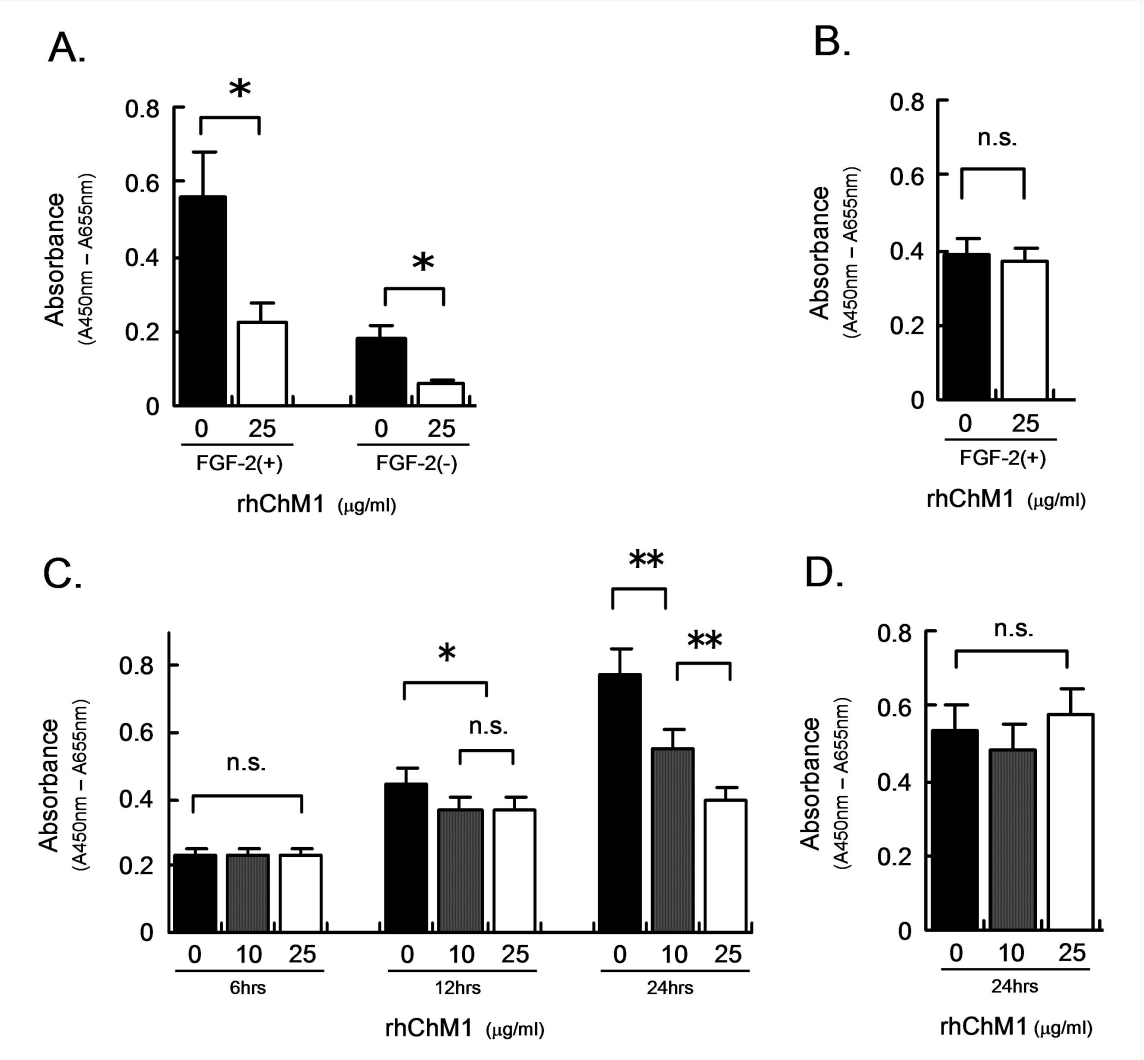
**A BrdU incorporation assay indicated that rhChM1 directly suppressed DNA synthesis of HUVECs (A) and HepG2 cells (C) cultured on plates**. rhChM1 significantly inhibited DNA synthesis in HUVECs, with or without FGF-2 (A), but did not in NHDFs with FGF-2 (B). HepG2 and HeLa cells were cultured in growth medium for 36 hrs, and then in fresh growth medium containing rhChM1 for the next period as indicated. rhChM1 inhibited DNA synthesis of HepG2 in a dose-dependent manner (C), but had no effect on HeLa cells (D). Each column and bar represent the mean and SD (n = 4). *: p < 0.05; **: p < 0.001; NS: not significant.

The effect of the rhChM1 protein on DNA synthesis was examined using a BrdU incorporation assay. At a concentration of 25 μg/ml, rhChM1 suppressed BrdU uptake in HUVECs with or without FGF-2 (Figure 1A), but not in NHDFs (Figure 1B). The presence of FGF-2 in the culture medium increased the uptake of BrdU and amplified the effect of ChM1 in HUVECs (Figure 1A). These results are consistent with those reported previously [5,8,32]. Recombinant human ChM1 also suppressed BrdU uptake by HepG2 cells. This effect first became evident at 12 hours after the addition of ChM1 and further increased at 24 hours in a dose-dependent manner (Figure 1C). In contrast, rhChM1 did not affect BrdU uptake by HeLa cells (Figure 1D). As our supply of rhChM1 was limited, we used adenovirus carrying ChM1 cDNA (Ad-ChM1) in subsequent experiments.

### Expression of human ChM1 protein induced by adenovirus vector

**Figure 2:**
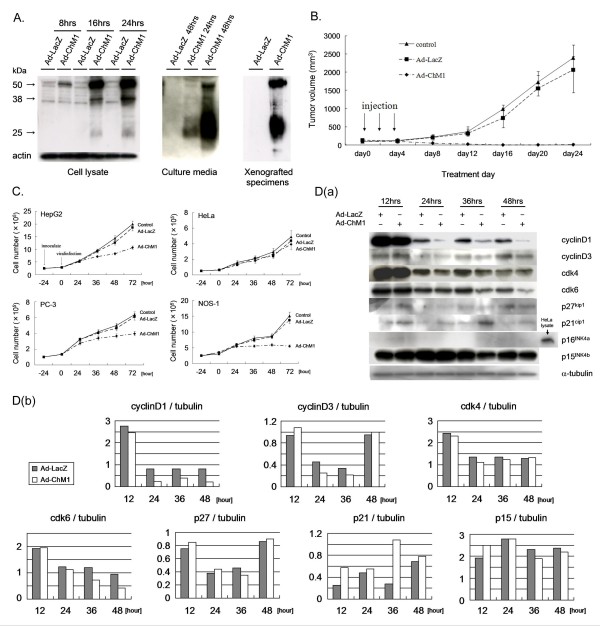
**The effect of ChM1 on growth of tumor cells cultured on plates**. A. Western blot analysis of the expression of transfected ChM1 in cells and xenografted tumors. ChM1 protein was detected in cells 8 hrs after transfection and increased in a time-dependent manner (left panel). The amount of protein secreted into the medium also increased in a time-dependent manner (middle panel). The protein was detected in xenografted tumor specimens harvested 48 hrs after transfection (right panel). The diffuse 25 kDa band corresponds to the monomer and glycosylated forms of ChM1 and the bands at approximately 38 kDa and 50 kDa correspond to the precursor and dimerized forms of ChM1, respectively. B. The effect of ChM1 on the growth of implanted HepG2 cells in athymic mice. Adenoviral gene therapy was initiated when tumors attained a volume of 150 mm^3^. ChM1 caused a complete suppression of tumor growth. Each point and bar represents the mean and SD (n = 6). C. ChM1 growth suppression of tumor cells cultured on plates. The inhibitory effect of ChM1 was observed 36 hrs after transfection in HepG2, PC-3 and NOS-1, but not in HeLa cells (n = 4). Each point and bar represents the mean and SD (n = 4). D. ChM1 alters the levels of cell cycle-related proteins in HepG2 cells cultured on plates. (a) Western blot analysis demonstrated that ChM1 altered the levels of some of cell cycle-related proteins. (b) Corresponding densitometry analysis showed that ChM1 significantly decreased from 24 hours, while cdk6 protein decreased from 36 hours, after adenoviral infection. The level of cyclin D3 protein was significantly decreased by ChM1 at 24 hours and 36 hours. In contrast, ChM1 significantly increased the level of p21^cip1^, a cdk inhibitor, at 12 hours and 36 hours.

Cells were transfected with Ad-ChM1 and cultured. Cell lysates and culture medium supernatants were analyzed for ChM1 protein by western blotting. It has been reported that ChM1 is first produced as a 38 kDa precursor that is then digested by furin to form a 25 kDa monomer [32,33]. Two monomers form a dimer that is secreted and is then localized on the plasma membrane as a monomer with a modification of the sugar chains (approximately 25 kDa). In our study, we obtained data consistent with those expected of ChM1; an example of a western blot is shown in Figure 2A.

### Ad-ChM1 inhibits tumor growth in vivo

BALB/c athymic mice were inoculated with HepG2 cells and the consequent tumors were allowed to grow to a volume of 150 mm^3^. In mice injected with vehicle only or Ad-LacZ, the tumors continued to grow and showed a 15-fold increase (150 mm^3 ^vs 2395.5 mm^3^) in size by day 24 (Figure 2B). In contrast, Ad-ChM1 injection not only produced complete inhibition of tumor growth, but also diminished tumor size significantly (150 mm^3 ^vs 12 mm^3^; Figure 2B). Tumors actually disappeared completely in 4 of the 6 mice injected with Ad-ChM1. These data are in good agreement with our previous observations.

### Ad-ChM1 affects the growth of some tumor cell lines in vitro

We next examined the effect of Ad-ChM1 on various tumor cell lines *in vitro*. Transfection with Ad-ChM1 significantly reduced cell growth in HepG2, PC-3 and NOS-1 cell cultures at 36 hours and thereafter (Figure 2C) compared to the vehicle- or Ad-LacZ treated groups, but did not affect the growth of HeLa cells. Trypan blue staining revealed that in all cell lines, most of the cells on each culture plate were viable at 48 and 72 hours, although there was a slight decrease in the proportion of viable cells at 72 hours (data not shown). Infection efficiency was adjusted by setting the MOI to ensure that more than 80% of the Ad-LacZ-treated cells were stained in an X-gal assay (data not shown).

### ChM1 alters expression of cell cycle-related proteins in HepG2 cultured on plates

To investigate the mechanism of ChM1-induced suppression of tumor cell growth, we examined the expression levels of cell cycle-related proteins in HepG2 cells *in vitro *by western blotting analysis. As depicted in Figure 2D(a), Ad-ChM1 altered the levels of some of cell cycle-related proteins by 36 hours after infection and the effect was maintained up to 48 hours. In a corresponding plot of the densitometry analysis shown in Figure 2D(b), the levels of cyclin D1, cyclin D3, and cdk6 were significantly decreased by Ad-ChM1. In contrast, Ad-ChM1 caused up-regulation of p21^cip1^, a cdk inhibitor, at 12 hours and 36 hours. Results of repeated experiments were similar, but the signal contrasts of those proteins were different due to exposure conditions of each membrane. RT-PCR analysis demonstrated that the levels of gene expression of these cell cycle-related proteins were unaffected by viral infection (data not shown).

### ChM1 suppresses anchorage-independent growth of HepG2 and HeLa cells

**Figure 3:**
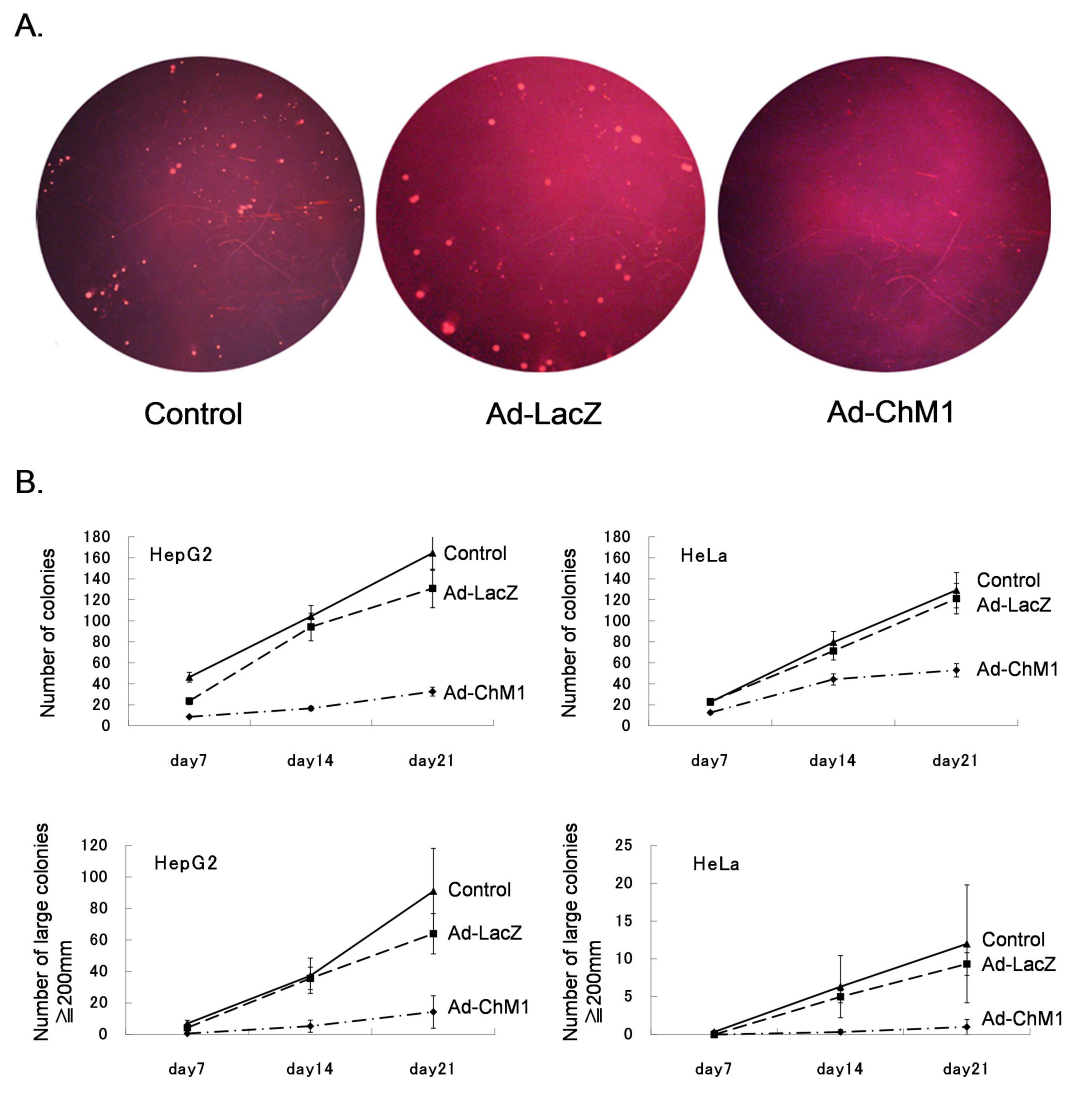
**The effect of ChM1 on colony formation by tumor cells**. A. HepG2 cells with or without adenoviral infection cultured in agarose gel for 21 days. On day 21, HepG2 cells infected with Ad-ChM1 formed fewer colonies, compared to cells infected with Ad-LacZ and to non-treated cells. Image magnification ×8. B. Effect of ChM1 on colony formation by HepG2 and HeLa cells. ChM1 not only reduced the number of colonies but also the size of colonies formed by HepG2 and HeLa cells. The effect of ChM1 on HeLa cells observed here is in stark contrast to its ineffectiveness on cells cultured on plates (Figure 2C). This results suggest that ChM1 suppresses anchorage-independent growth. Each point and bar represents the mean and SD (n = 3).

We next examined the effect of ChM1 on anchorage-independent growth, which is a hallmark of tumor cells. At 6 hours after infection with Ad-ChM1, HepG2 and HeLa cells were detached from the plates, suspended in soft agarose gel and a colony formation assay was carried out. Colonies were first detected at 4 days in control cultures (Figure 3A) and continued to increase in size with time (Figure 3B). Ad-ChM1 infection markedly suppressed the total number of colonies and of large colonies in the HepG2 cell cultures. These data are consistent with those shown in Figure 1C and 2C that were obtained from cells grown on plates. Ad-ChM1 markedly suppressed the number of colonies in HeLa cell cultures. This result is in sharp contrast to the data obtained from culturing HeLa cells on plates (Figures 1D and 2C). Ad-LacZ infection slightly reduced the number of colonies, and this reduction was significant for HepG2 cells at 21 days (Figure 3B). These data clearly demonstrate that ChM1 is capable of suppressing anchorage-independent growth of HepG2 and HeLa cells, a result that is consistent with its *in vivo *anti-tumor effect (Figure 2B and ref [7]). ChM1 was more effective in HepG2 than HeLa cells, and the reduction in total colony number was 80% vs 50% at day 14 and 87.5% vs 70% at day 21, respectively.

### Effect of ChM1 on downstream molecules of the extracellular matrix-integrin signaling pathway

**Figure 4:**
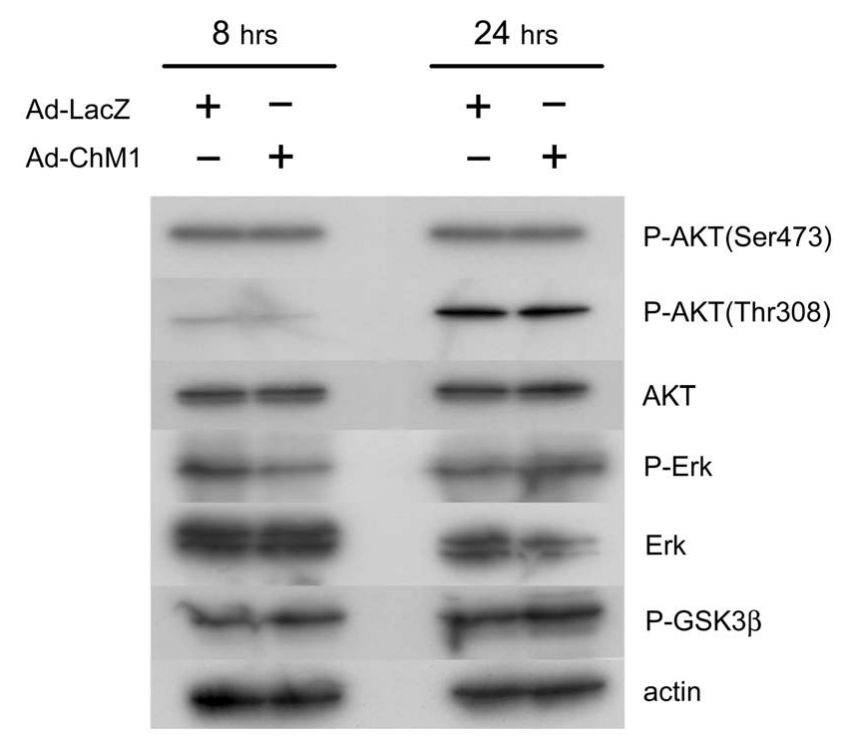
**The effect of ChM1 on the downstream pathway of extracellular matrix-integrin signaling**. Western blots showing phosphorylation levels of Erk, Akt, and GSK3β, the downstream molecules of the extracellular matrix-integrin signaling pathway. ChM1 had no effect on phosphorylation levels of these proteins at 8 and 24 hours after adenovirus infection.

As described above, we demonstrated that ChM1 directly suppressed anchorage-independent tumor cell growth. The mechanism of this action, however, was difficult to elucidate, since neither the receptors nor the downstream signaling molecules have been identified. Anchorage-dependent signaling utilizes integrins and their downstream signaling pathway, which converges with one of the anchorage-independent pathways that includes signaling molecules such as Akt, Erk, and GSK3β [10,20-22]. We examined this pathway first using western blot analysis and found that phosphorylation of Akt, Erk and GSK3β was unaffected (Figure 4).

### ChM1 modulates the STAT pathway

**Figure 5:**
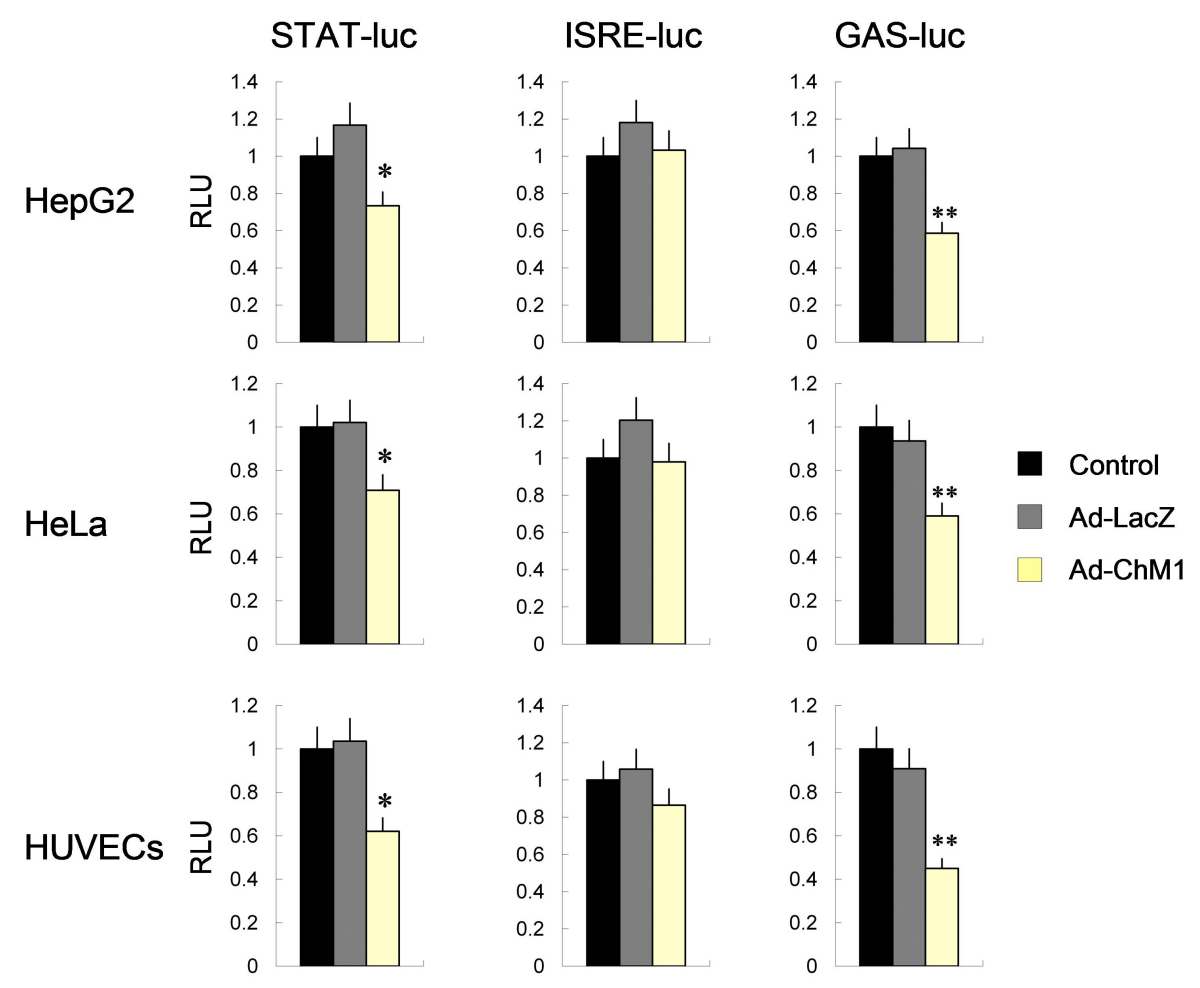
**ChM1 modulates the STAT pathway**. The effect of ChM1 on signal transduction pathways analyzed using a luciferase assay. ChM1 inhibited transcriptional activation through the STAT-luc by 30% in HepG2 and HeLa cells and by 40% in HUVECs. ChM1 inhibited transcriptional activation through the GAS-luc by 40% in HepG2 and HeLa cells and by 60% in HUVECs. In contrast, ChM1 did not affect transcriptional activity through the ISRE-luc in these cell lines. Each column and bar represent a mean and SD (n = 4). (*: p < 0.05; **: p < 0.001).

The luciferase reporter assay demonstrated that Ad-ChM1 suppressed the promoter activity of STAT-luc and GAS-luc, but did not affect ISRE-luc promoter activity in HepG2, HeLa and HUVECs cultured on plates (Figure 5). The three cell types showed similar patterns of response to Ad-ChM1. As described above, the growth of HeLa cells cultured on plates was not affected by ChM1 (Figure 2C). Nevertheless, the STAT pathway was suppressed by ChM1 in HeLa cells in a similar manner to HepG2 cells and HUVECs (Figure 5), indicating that ChM1 caused growth inhibition.

## Discussion

Previously, we reported that rhChM1 inhibits growth of chondrosarcomas *in vivo *[7], but our understanding at that time was that the mechanism of the inhibitory effect was solely due to the anti-angiogenic activity of ChM1. In this study, we demonstrated that ChM1 has *in vivo *and *in vitro *anti-tumor activity against the hepatocyte tumor cells, HepG2, and that the effect is due not only to its anti-angiogenic activity but also to direct inhibition of tumor cell growth. Moreover, our results showed that the Jak/STAT signaling pathway is one of the targets of ChM1 action.

Monotherapy with the anti-VEGF antibody, bevacizmab, or an endogenous anti-angiogenic agent such as endostatin caused only a moderate suppression of tumor growth compared with a combined therapy with a cytotoxic agent [2,3]. These results indicate that a molecule with both anti-angiogenic and direct cytotoxic activity should be superior for the treatment of patients with malignant tumors. In this regard, our finding that ChM1 has the ability not only to inhibit angiogenesis, but also to inhibit tumor growth is of interest. ChM1 is the first example of an endogenous molecule with both anti-angiogenic and cytotoxic activities and our results suggest that this molecule warrants further *in vivo *study in the future.

In addition to its anti-angiogenic activity, ChM1 is also known to have chondrocyte modulating activity [4,6], bone remodeling activity [34], and T-cell suppressing activity [9]. In particular, ChM1 also promotes the anchorage-independent growth of chondrocytes [6]. Anchorage-independent growth is a characteristic of non-adherent cells, including oncocytes [10], chondrocytes [11-14], and hemocytes [15,16]. As is shown in Figure 2, the growth of HeLa cells cultured on plates was not affected by ChM1, whereas the growth of HepG2, PC-3 and NOS-1 cells was significantly suppressed. In contrast, the growth of HeLa cells cultured in soft agarose gel was suppressed by ChM1 in a similar fashion to HepG2 cells, although the effect on HeLa cells was slightly less (Figure 3B). These data indicate that ChM1 inhibits the anchorage-independent growth of tumor cells.

Moreover, our observations also provide some suggestion as to why the results of plate culture produces conflicted with those obtained from soft agarose gel culture. The luciferase reporter assay, carried out on cells cultured on plates, demonstrated that ChM1 suppressed the promoter activity of STAT-luc and GAS-luc in HeLa cells to a similar extent as in HepG2 cells and HUVECs. This appears to be inconsistent with the fact that ChM1 inhibited the growth of HepG2, but not HeLa cells cultured on plates. When the basal promoter activities of STAT-luc and GAS-luc were examined, however, HepG2 cells were found to have the highest levels, followed by HUVECs. In contrast, the basal levels of HeLa cells were much lower than that of the other cells(data not shown). Thus, the basal promoter activities of STAT-luc and GAS-luc may be negligible in HeLa cells. Taken together with the observation that the growth of HeLa cells on plates was not affected by ChM1, these data suggest that ChM1 inhibits the anchorage-independent growth of cells, and, therefore, its effect on cells cultured in soft agarose gel may be achieved by inhibition of the Jak/STAT pathway. When cells are cultured on plates, however, the effect of ChM1 on cell growth varies depending upon the degree to which the cells rely on the Jak/STAT pathway for growth. Thus, the growth of HeLa cells cultured on plates was unaffected by ChM1, since anchorage-dependent growth plus the anchorage-independent non-Jak/STAT pathway may contribute to growth. This explanation is consistent with our observation that phosphorylation of Akt, Erk and GSK3β, signaling molecules downstream of integrin-mediated signal transduction [10,20-22] and the anchorage-independent non-Jak/STAT pathway, was not affected by ChM1.

However, it is unclear how ChM1 activates intracellular signaling pathways and whether there are specific receptors for ChM1. We have shown that ChM1 suppresses the promoter activity of STAT-luc and GAS-luc, but not of ISRE-luc. ChM1 may act through one or more of the following mechanisms: 1) by recruiting protein tyrosine phosphatase family members such as SHP which inactivate Jak; 2) by recruiting SOCS and/or PIAS to degrade STAT dimers; or 3) by directly or indirectly inhibiting cofactors that form complexes with STAT dimers [24,35]. Obviously, further study is required to examine these mechanisms.

The cytotoxic action of ChM1 may be due to growth arrest, apoptosis or a combination of both. Our results strongly indicate that ChM1 mainly causes growth arrest. First, ChM1 inhibited DNA synthesis (Figure 1) and suppressed cell proliferation during culture on plates (Figure 2C), as well as in soft agar (Figure 3). Second, ChM1 down-regulated proteins such as cyclinD1, cyclinD3, and cdk6 that promote cell division, and up-regulated cdk inhibitors such as p21^cip1 ^(Figure 2D). Third, cells treated with ChM1 were mostly viable and the number of apoptotic cells was negligible throughout the culture period (data not shown). Taken together, these data suggest that the cytotoxic effect of ChM1 is mainly due to cell cycle arrest, and that apoptosis does not play an important role, if any. To some extent, our data contradict a recent observation that ChM1 induces apoptosis of vascular endothelial cells [36]. The reasons for this inconsistency are not clear at present, but may be due to the use of different cell types and/or experimental conditions in the two studies. Possibly, the effect of ChM1 varies between cell types depending on differences in cell cycle regulation and the balance of signaling pathways that can be directly or indirectly affected by the protein (see discussion above). Our study suggests that ChM1 suppresses the growth of tumor cells by directly arresting the cell cycle and that apoptosis does not play a major role.

## Conclusion

We have demonstrated that ChM1 produces an anti-tumor effect not only by inhibiting angiogenesis but also by inducing growth arrest of tumor cells, and by directly suppressing the proliferation of tumor cells in an anchorage-independent manner. However, ChM1 did not alter the phosphorylation of the downstream molecules at which the signaling pathways through receptors for growth factors and cytokines converge with the anchorage-dependent pathway. The mechanism of the induced growth arrest appears to involve the anchorage-independent Jak/STAT pathway.

ChM1 is the first example of an endogenous molecule that possesses two different anti-tumor actions. Our results clearly indicate that this molecule warrants further study *in vivo*.

## Competing interests

The authors declare that they have no competing interests.

## Authors' contributions

HM and HK conceived the project and contributed to its design, HM mainly performed the experiments and wrote/reviewed/edited the manuscript, HK performed adenovirus preparation and animal experiments, and co-wrote part of the manuscript. TY and OI supervised the project and contributed to its design, analysis and interpretation of the data. MMA supported cell culture and western blotting analysis, interpreted the data, for which they are due special thanks. TH, HK, HY supervised and supported the project particularly aspects involving ChM1. NK contributed to amplification of the adenovirus and suggested the experiment. AO contributed to the finance of the project. NE contributed to the finance of the project, conceived and supervised the project. HK contributed to organization of the project, analyzed and interpreted the data and co-wrote the manuscript. All authors read and approved the final manuscript.

## Pre-publication history

The pre-publication history for this paper can be accessed here:

http://www.biomedcentral.com/1471-2407/9/166/prepub
